# The Johns Hopkins Hospital Reports

**Published:** 1896-07

**Authors:** 


The Johns Hopkins Hospital Reports. Volume IV., No. 9. Report in Pathology. Deciduoma Malignum. By J. Whitridge Williams, M. D. Baltimore : The Johns Hopkins Press. 1895.

This report is devoted entirely to a case of deciduoma malignum and is the first authentic case yet reported in America or England, although many cases have been reported on the continent. Sänger, in 1888, first reported a case of this kind in a 23-year-old woman, who aborted in the eighth week and died seven months later with four large, soft, spongy, reddish tumors in the uterine wall and metastasis of the same character in the lungs, diaphragm, tenth rib and right iliac fossa. Microscopically the neoplasm appeared to be a malignant metastasising deciduoma, belonging to the sarcoma group.

The case in question resembles closely that of Sänger's in its gross appearance and clinical history, but differs very essentially from it in its firmer structure. Both present the same alveolar structure and are characterised by their markedly hemorrhage nature; but Sänger's new growth is composed of decidual cells while Williams's is made up of allular elements whose significance is not beyond all question.

The author then discusses carefully the nature and histogenesis of the mass and compares it with the various new growths which have been described as malignant deciduoma. The frequency, clinical history, etiology, diagnosis and treatment are also considered.

A complete bibliography and a double-page plate accompany the article. W. C. K.

